# Unveiling the Mystery of Horseshoe Lung: A Case Report

**DOI:** 10.7759/cureus.80963

**Published:** 2025-03-21

**Authors:** Mehr Nisa, Andrina Panicker, Shakeel Ahmed, Irfan Ul Haq

**Affiliations:** 1 Family Medicine, Primary Health Care Corporation, Doha, QAT; 2 Medicine, All India Institute of Medical Sciences, New Delhi, New Delhi, IND; 3 Medicine, Hamad Medical Corporation, Doha, QAT; 4 Pulmonology, Hamad Medical Corporation, Doha, QAT

**Keywords:** adult scimitar syndrome, clinical embryology, congenital lung lesions, diagnostic ct imaging, horseshoe lung

## Abstract

Horseshoe lung is a rare congenital anomaly where the lower lung segments fuse behind the heart, posing diagnostic challenges that require advanced imaging. In a case involving a 33-year-old male presenting to primary care with left-sided chest pain, an abnormal chest X-ray revealed reduced lung volume and a cystic structure on the left. A CT scan confirmed a small, hypoplastic left lung and compensatory right lung expansion and fusion, leading to a diagnosis of horseshoe lung. The patient, with no significant medical history and normal vital signs, was asymptomatic when seen in the pulmonology clinic and scheduled for routine follow-up. First identified in 1962, horseshoe lung can be confused with other conditions due to its overlapping features. The exact embryological origins are unclear, but advanced imaging such as multidetector computed tomography (MDCT) is essential for accurate diagnosis. Effective management of horseshoe lung requires a multidisciplinary approach and advanced imaging for precise diagnosis and treatment. Further research is needed to understand its embryological origins.

## Introduction

Horseshoe lung, an exceptionally rare congenital pulmonary anomaly, is characterised by the fusion of the lower lung segments behind the heart and in front of the oesophagus, thoracic aorta, and spine [1]. First identified in 1962, this condition continues to intrigue medical professionals due to its infrequent occurrence and potential for diagnostic overlap with other pulmonary and cardiovascular diseases [2]. It is often associated with underdevelopment of one lung and abnormal pulmonary venous return to the systemic veins. Other related anomalies, especially cardiovascular ones, have also been documented [3].

While the existing literature has described cases of horseshoe lung, often diagnosed in symptomatic individuals or associated with other congenital anomalies, this case report presents a noteworthy instance of incidental discovery in an asymptomatic 33-year-old male [4,5]. This is significant as most cases are diagnosed earlier in life due to the presence of symptoms [1,6]. Furthermore, this case highlights the rare association of horseshoe lung with left lung hypoplasia, a less common finding compared to the more frequently reported right lung hypoplasia [4]. This underscores the variable clinical spectrum of horseshoe lung with its embryological origins remaining unclear. Treatment and prognosis generally depend on the presence of other concomitant anomalies and the severity of symptoms.

## Case presentation

A 33-year-old male was referred to the chest clinic following an abnormal chest X-ray. He initially presented to a primary health center with left-sided chest pain, where a chest X-ray was performed. At his appointment in the chest clinic, he was asymptomatic from a respiratory perspective. He had no prior history of chest illnesses and no family history of lung disease. He had a smoking history of 10 pack years.

**Figure 1:**
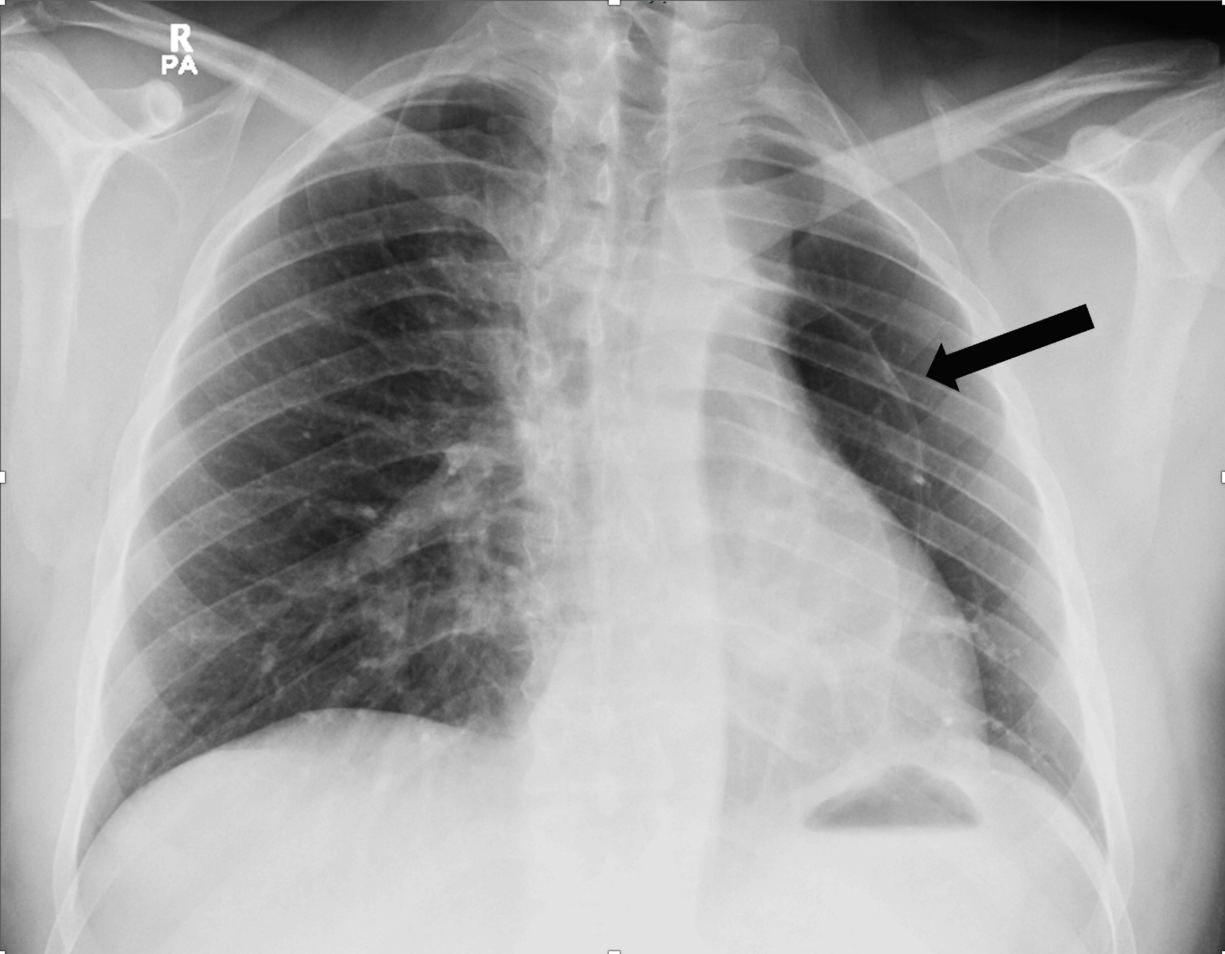
Chest X-ray shows reduced volume of the left lung with a cystic structure at the left lung hilum (arrow).

On examination, his vital signs were normal, with a SpO_2_ of 99% on room air. His chest was clear to auscultation, and there was no evidence of clubbing. The chest X-ray revealed a small left lung volume and a cystic structure at the left hilum (Figure 1).

**Figure 2:**
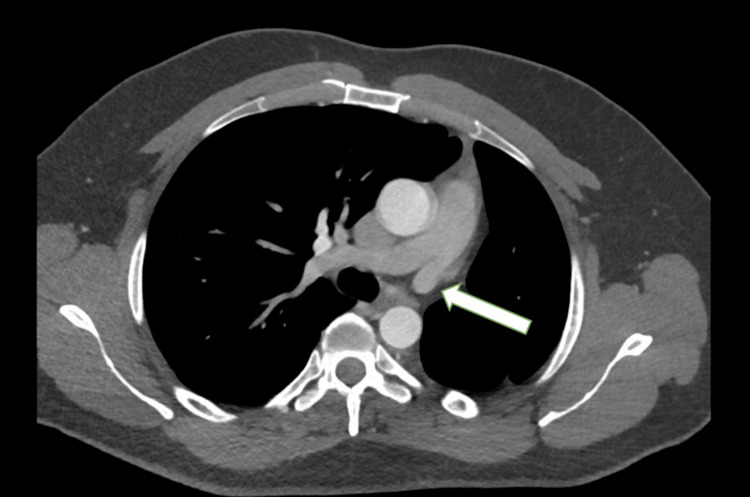
CT chest (mediastinal window) showing a hypoplastic left pulmonary artery (arrow).

**Figure 3:**
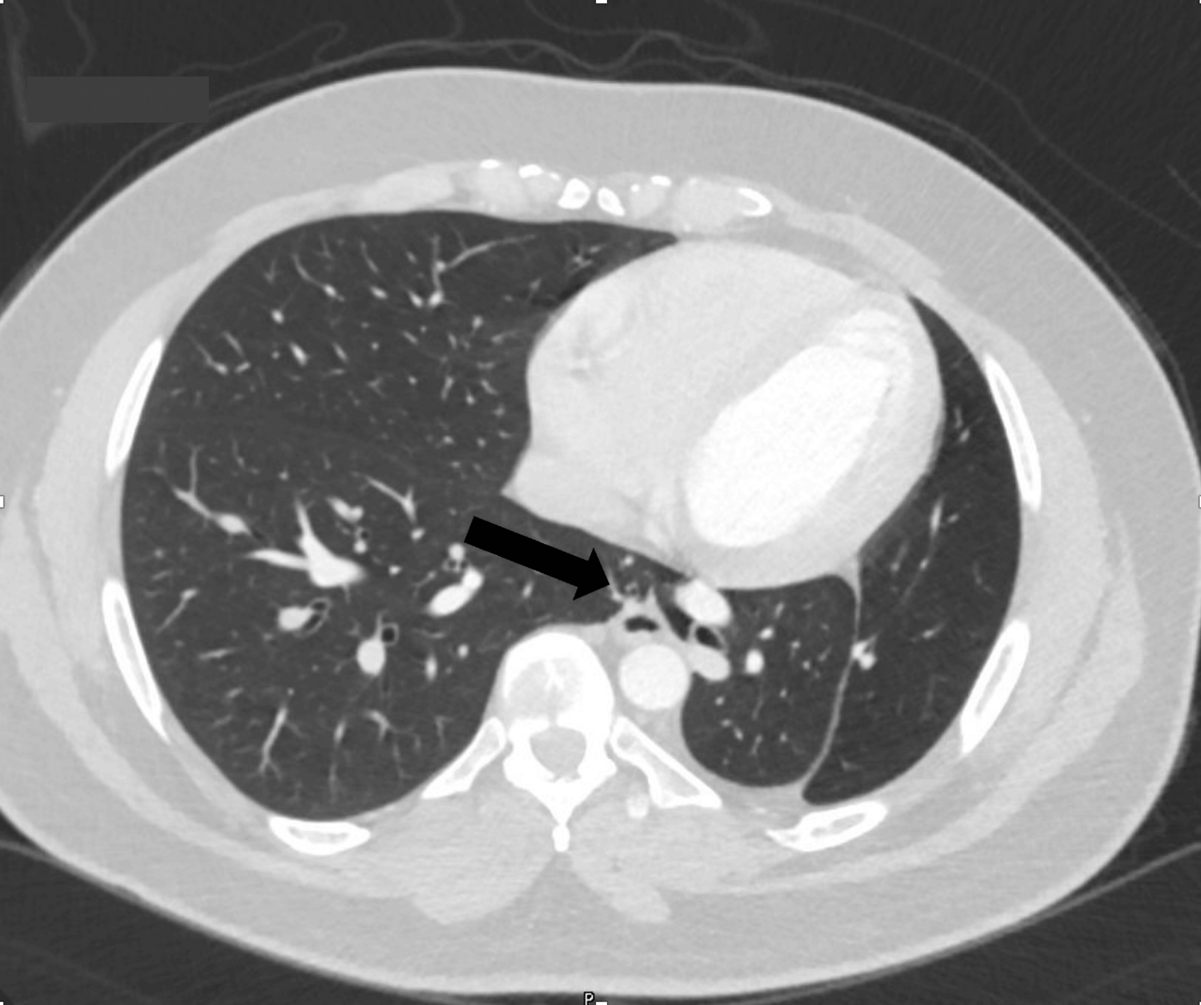
CT chest demonstrates reduced left lung volume with herniation of the right lung into the left hemithorax, positioned behind the heart and anterior to the descending aorta (arrow).

**Figure 4:**
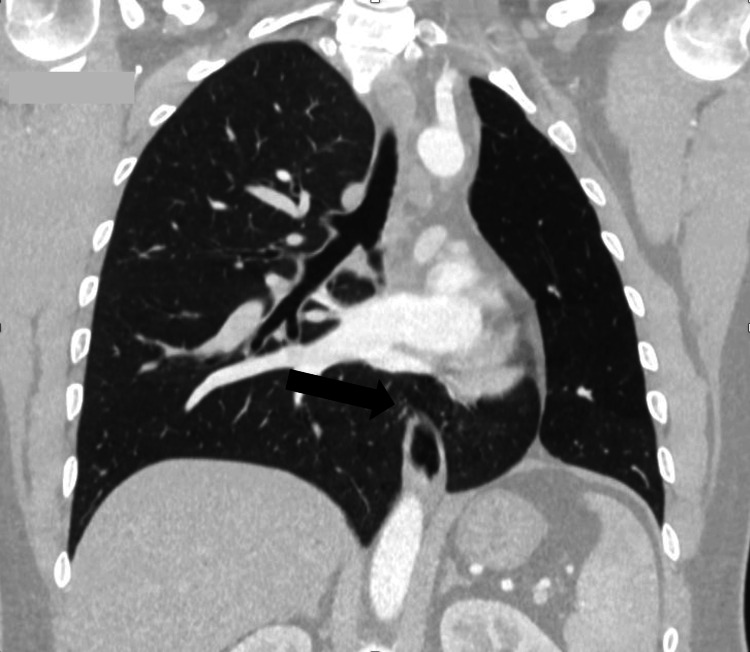
Multiplanar reconstruction (MPR) image shows compensatory expansion of the right lung, with the lower lobe extending across the midline into the medial aspect of the left hemithorax (arrow).

**Figure 5:**
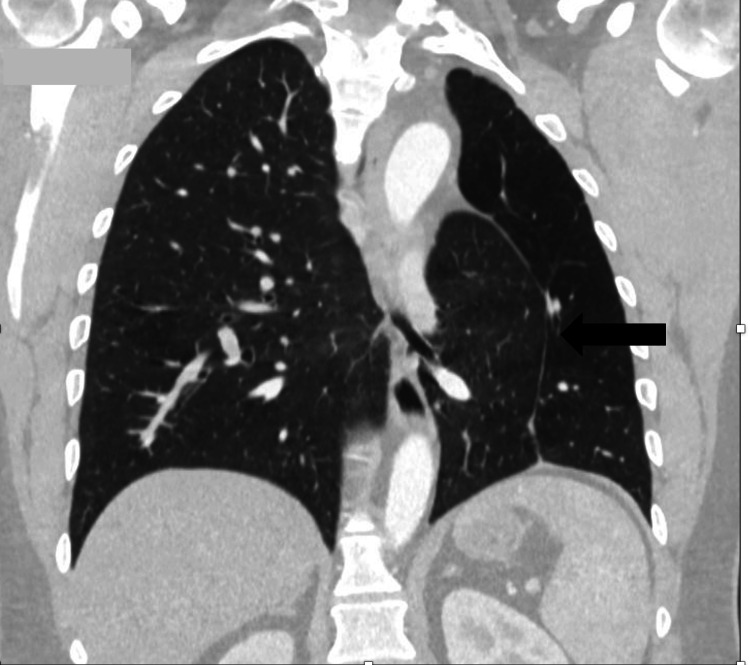
Multiplanar reconstruction (MPR) image shows herniation of the right lung into the left hemithorax, creating a misleading appearance of a cystic structure at the left hilum (arrow).

A contrast-enhanced CT scan of the chest demonstrated a marked reduction in the volume of the left hemithorax, with a small, hypoplastic left lung and a hypoplastic left pulmonary artery. There was significant compensatory expansion of the right lung, with the lower lobe herniating behind the heart and anterior to the descending aorta, crossing the midline into the medial aspect of the left hemithorax. This herniation filled the space between the mediastinum and the hypoplastic left lung. Additionally, there was marked leftward displacement of the mediastinum. The left pulmonary arteries and veins were moderately atrophied and hypoplastic, while the right lung was enlarged and showed compensatory hypertrophy. These findings are consistent with a diagnosis of horseshoe lung (Figures 2-5).

An echocardiogram revealed a normal ejection fraction, normal cardiac chambers, no valvular abnormalities, and no evidence of pulmonary hypertension. As the patient was asymptomatic, he was reassured regarding the findings and scheduled for routine follow-up at the chest clinic.

## Discussion

Horseshoe lung, an exceptionally rare congenital pulmonary anomaly, continues to intrigue medical professionals and researchers alike. This condition, first identified in 1962 by Spencer, presents a unique fusion of the lower lung segments behind the heart, positioned anterior to vital structures such as the aorta, oesophagus, and spinal column with a unique characteristic correlation with cardiac dextroversion and anomalous arterial supply to the right hypoplastic pulmonary constituent [1-3]. Despite its infrequency, horseshoe lung represents a complex medical challenge, demanding a deeper understanding of its embryological origins and intricate clinical manifestations.

The embryological origins of horseshoe lung remain unclear. Several theories exist regarding this anomaly. Thuong Vu et al. suggested that it may result from either a fusion process in the lung parenchyma or the failure of the splanchnic mesoderm to separate during the fourth week of gestation, leading to a connection between the lungs and the pleural cavity [7]. This eventuality leads to an unusual connection between the lungs and the pleural cavity, forming the distinct horseshoe-shaped structure observed in affected individuals [2].

Horseshoe lung can be classified into three groups based on the presence of pleura: fusion of the lungs without the pleura, presence of two pleural layers between the fused lung tissues, and the presence of four pleural layers between the fused lung tissues, each with its own visceral and parietal pleural envelopes [8].

This classification helps in understanding horseshoe lung in relation to its clinical symptoms. We believe our case falls into the second group. Although it may be asymptomatic and incidentally discovered, as in our case where the initial symptom of chest pain was likely unrelated, it can, however, present with respiratory distress, pneumonia, recurrent pulmonary infections, or symptoms of hypertension [4-6].

Unilateral lung hypoplasia, primarily on the right side, is a hallmark feature, observed in approximately 80% of cases drawing a parallel with another intriguing anomaly called scimitar syndrome [4]. Left lung hypoplasia in association with horseshoe lung is a rare occurrence and was seen in our case [9].

Diagnosing horseshoe lung presents formidable challenges, primarily due to the overlap of its clinical features with other pulmonary and cardiovascular conditions. Traditional diagnostic methods, such as plain X-rays, may provide initial insights, revealing characteristic signs like descending pulmonary vein, hypoplastic lung, and dextroposition. However, these signs might be obscured by the cardiac shadow, necessitating more advanced imaging techniques.

Echocardiography, magnetic resonance imaging (MRI), and CT angiography are essential diagnostic tools. These advanced imaging techniques provide detailed insights into the anatomical complexities of horseshoe lungs, allowing for a thorough assessment of the condition. Invasive procedures such as cardiac catheterization can play a vital role in confirming the diagnosis, identifying the route of anomalous pulmonary venous drainage, and evaluating the pressure and structure of the pulmonary artery vasculature [1,10].

Among the various diagnostic methods, multidetector computed tomography (MDCT) stands out as a preferred noninvasive technique. MDCT offers exceptional clarity and precision in imaging, providing detailed visualizations of the complex malformations associated with horseshoe lungs. Its capability to capture intricate anatomical details assists healthcare professionals in making accurate diagnoses and developing effective treatment strategies [11].

The significance of MDCT extends beyond mere diagnosis; it is instrumental in understanding the severity and unique features of horseshoe lungs. This imaging technology enables clinicians to clearly define the anomaly's boundaries, identify associated vascular structures and anomalies, and evaluate the extent of lung hypoplasia. Such comprehensive assessments form the basis for personalized treatment plans, ultimately improving patient outcomes and quality of life.

Horseshoe lung can have significant clinical implications due to the associated cardiac or vascular anomalies such as atrial septal defect (ASD), ventricular septal defect (VSD), patent ductus arteriosus (PDA), tetralogy of Fallot (TOF), and absent pulmonary artery, as well as pulmonary abnormalities like bronchoesophageal fistulas, requiring careful evaluation. Most cases are diagnosed at an earlier age than our case due to symptoms [1,6]. Although familial cardiac anomalies associated with horseshoe lung are rare, interesting gender-related patterns have been observed. Studies show a higher incidence of horseshoe lung in females than in males, adding complexity to understanding this anomaly [5]. Further research is needed to uncover the genetic and hormonal factors contributing to this gender disparity [12].

The study of horseshoe lung lies at the intersection of embryology, radiology, and clinical medicine. Ongoing collaborative research is essential to unravel the mysteries of this anomaly. Exploring its embryological origins, genetic underpinnings, and potential environmental influences are crucial areas for further investigation.

Collaboration among multidisciplinary healthcare professionals including pulmonologists, cardiologists, radiologists, and geneticists will not only facilitate accurate diagnosis but also pave the way for innovative therapeutic interventions and genetic counselling for affected individuals and their families.

## Conclusions

In conclusion, although rare, horseshoe lung is a fascinating area of study within congenital pulmonary anomalies. Its complex clinical presentations, diagnostic challenges, and notable gender disparities highlight the need for a comprehensive approach to research and clinical management. Advanced imaging techniques, especially MDCT, have proven invaluable, offering detailed insights into the anomaly's anatomical intricacies. The mysteries of horseshoe lung should encourage researchers and clinicians to explore its origins, clinical manifestations, and potential treatments further, uncovering its secrets to enhance diagnostics, develop targeted treatments, and improve outcomes for those affected by this rare congenital anomaly.
